# Effectiveness of tigecycline-based versus colistin- based therapy for treatment of pneumonia caused by multidrug-resistant *Acinetobacter baumannii* in a critical setting: a matched cohort analysis

**DOI:** 10.1186/1471-2334-14-102

**Published:** 2014-02-24

**Authors:** Yu-Chung Chuang, Chien-Yu Cheng, Wang-Huei Sheng, Hsin-Yun Sun, Jann-Tay Wang, Yee-Chun Chen, Shan-Chwen Chang

**Affiliations:** 1 Department of Internal Medicine, National Taiwan University Hospital, No. 7 Chung-Shan South Road, Taipei 100, Taiwan; 2 Graduate Institute of Clinical Medicine, College of Medicine, National Taiwan University, Taipei Taiwan; 3 Department of Internal Medicine, Tao-Yuan General Hospital, Tao-Yuan, Taiwan; 4 Department of Medicine, National Taiwan University College of Medicine, Taipei, Taiwan

**Keywords:** *Acinetobacter baumannii*, Pneumonia, Colistin, Tigecycline, Mortality, Nephrotoxicity

## Abstract

**Background:**

Colistin and tigecycline have both been shown good *in vitro* activity among multi-drug resistant *Acinetobacter baumannii* (MDRAB). A comparative study of colistin versus tigecycline for MDRAB pneumonia is lacking.

**Methods:**

The study enrolled adults with MDRAB pneumonia admitted to intensive care units at a referral medical center during 2009–2010. Since there were no standardized minimum inhibitory concentration (MIC) interpretation criteria of tigecycline against *A. baumannii*, MIC of tigecycline was not routinely tested at our hospital. During the study periods, MIC of colistin was not routinely tested also. We consider both colistin and tigecycline as definite treatments of MDRAB pneumonia. Patients who received tigecycline were selected as potential controls for those who had received colistin. We performed a propensity score analysis, by considering the criteria of age, gender, underlying diseases, and disease severity, in order to match and equalize potential prognostic factors and severity in the two groups.

**Results:**

A total of 294 adults with MDRAB pneumonia were enrolled, including 119 who received colistin and 175 who received tigecycline. We matched 84 adults who received colistin with an equal number of controls who received tigecycline. The two well matched cohorts share similar characteristics: the propensity scores are colistin: 0.37 vs. tigecycline: 0.37, (*P* = .97); baseline creatinine (1.70 vs. 1.81, *P* = .50), and the APACHE II score (21.6 vs. 22.0, *P* = .99). The tigecycline group has an excess mortality of 16.7% (60.7% vs. 44%, 95% confidence interval 0.9% – 32.4%, *P* = .04). The excess mortality of tigecycline is significant only among those with MIC >2 μg/mL (10/12 vs. 37/84, *P* = .01), but not for those with MIC ≦ 2 μg/mL (4/10 vs. 37/84, *P* = .81).

**Conclusions:**

Our data disfavors the use of tigecycline-based treatment in treating MDRAB pneumonia when tigecycline and colistin susceptibilities are unknown, since choosing tigecycline-based treatment might result in higher mortality. The excess mortality of tigecycline-based group may be related to higher MIC of tigecycline (> 2 μg/mL). Choosing tigecycline empirically for treating MDRAB pneumonia in the critical setting should be cautious.

## Background

Healthcare-associated infections caused by *Acinetobacter baumannii* are increasing among patients in intensive care units (ICU) [1,2]. These infections are associated with a high mortality and a prolonged length of hospital stay [2]. Pneumonia is the most common clinical syndrome [3]. However, due to the emerging multi-drug resistance among *A. baumannii* isolates, the treatment choice for multi-drug resistant *A. baumannii* (MDRAB) related infection is limited [4].

Colistin and tigecycline have been shown to have good *in vitro* activity against *A. baumannii* pneumonia isolates, even in carbapenem-resistant isolates [5,6]. In fact, there have been successful experiences at using colistin in treating MDRAB pneumonia [7,8], and colistin is recommended as a treatment option for pneumonia caused by MDRAB [9]. However, poor pulmonary penetration [10] and renal toxicity are the major concerns [8,11]. Tigecycline also presents good *in vitro* activity against MDRAB isolates [6], and several studies have revealed acceptable clinical responses of tigecycline for MDRAB pneumonia [6,12,13]. Though there are no available minimum inhibitory concentration (MIC) interpretation breakpoints of tigecycline against *A. baumannii* according to the criteria of the Clinical and Laboratory Standards Institute (CLSI) or the European Committee on Antimicrobial Susceptibility Testing (EUCAST) [2], tigecycline have been suggested as an alternative drug of choice in treating MDRAB pneumonia [14]. In spite of all this, a comparative study of colistin versus tigecycline for MDRAB pneumonia is lacking [6,8,15]. The aim of the current study is to compares the effectiveness and the adverse effects of colistin-based versus tigecycline-based therapy in treating MDRAB pneumonia.

## Methods

### Study population

This is a retrospective study conducted among patients admitted to the ICU at the National Taiwan University Hospital (NTUH). NTUH is a 2,200**-**bed teaching hospital located in northern Taiwan that provides both primary and tertiary medical care. This study was approved by NTUH IRB committee.

Patients with MDRAB pneumonia and treated with colistin or tigecycline were enrolled in this study from January 2009 through December 2010. MDRAB was defined as *A. baumannii*, which showed non-susceptibility to ≥ 1 agent in ≥ 3 antimicrobial categories [16]. Pneumonia was defined based on a prospective surveillance by the microbiology lab and infection controls at NTUH [17]. A patient with a pneumonia must have either a new onset of purulent sputum, a change in the character of sputum, rales or dullness to percussion from a physical examination of the chest, chest radiographic examinations indicating new or progressive infiltrates, consolidation, cavitation, or pleural effusion and MDRAB isolated from the blood, bronchoscopic bronchial brushing, lavage or suitable sputum (defined as > 25 polymorphonuclear neutrophil and < 10 squamous epithelial cells per low power field). We used qualitative culture through the study. Ventilator-associated pneumonia (VAP) is defined as pneumonia developed 48 h or longer after mechanical ventilation is given by means of endotracheal intubation or tracheostomy. Patients are further classified by ventilator status: early-onset VAP (< 5 d of ventilation) or late-onset VAP (≥ 5 d of ventilation).

NTUH restricts the prescription of colistin or tigecycline to infectious disease physicians. In the circumstance of MDRAB infection, the prescriptions are limited to culture proven MDRAB, which showed non-susceptibility to ampicillin/sulbactam and the following anti-pseudomonas antibiotics, included cephalosporins, extended-spectrum penicillins, carbapenems, aminoglycosides, and fluoroquinolones. Since there were no MIC interpretation criteria of tigecycline against *A. baumannii*, MIC of tigecycline was not routinely tested at our hospital. During the study periods, MIC of colistin was not routinely tested also. Therefore, we consider both colistin and tigecycline as definite treatments of MDRAB pneumonia [2]. The dose of colistin (Colimycin injection, TTY Biopharm Co., Ltd., Taipei, Taiwan) was colistin base activity (CBA) 2.5–5 mg/kg/day in 2–3 divided doses (2 million units or 160 mg colistin methanesulfonate equivalent to 66.8 mg CBA). The dose of tigecycline was a 100 mg loading, followed by 50 mg every 12 hours.

This study excluded patients under the age of 18 years or with incomplete medical records. We also excluded patients with concomitant infection, such as methicillin resistant *Staphylococcus aureus* or *Pseudomonas aeruginosa*. When patients had multiple episodes of MDRAB pneumonia and were treated with colistin or tigecycline, only the first episode was included.

### Microbiological studies

This study identified *A. baumannii* by biochemical methods [18] and determined antimicrobial susceptibility by the disk diffusion method for gentamicin, amikacin, ciprofloxacin, levofloxacin, cefepime, ceftazidime, ticarcillin/clavulanate, meropenem, and ampicillin/sulbactam according to CLSI criteria [19]. We performed MICs of colistin by agar dilution methods according to CLSI criteria [19]. Lastly, MICs of tigecycline were determined by agar dilution methods (BBL, BD Diagnostic Systems, Sparks, MD), with isolates of MIC ≦ 2 μg/mL were considered as being susceptible [20].

### Data collection

We collected demographic data, underlying diseases, and the concomitant infections by a retrospective chart review, recorded regimens of antimicrobial combination, and evaluated the severity of pneumonia by the Acute Physiology and Chronic Health Evaluation (APACHE) II score [21]. The outcomes for evaluation are in-hospital mortality and nephrotoxicity. In patients with normal renal function, nephrotoxicity was defined as a doubling of baseline serum creatinine compared with treatment initiation, or a reduction in the calculated creatinine clearance (CLCr) of ≥ 50%. In patients with a pre-existing renal dysfunction, nephrotoxicity was defined as an increase of > 50% of the baseline creatinine level compared with the value at treatment initiation, a decrease of ≥ 20% of calculated serum CLCr from the baseline, or a decline in renal function that required renal replacement therapy [7,22]. Combination therapy was defined as at least 3 days of concurrent use of the antimicrobial agent other than colistin or tigecycline.

### Statistical analysis

We calculated the mean and standard deviation (SD) for continuous variables and percentages for categorical variables. We then compared the associations between the clinical presentations of colistin and tigecycline treatments using Student’s *t* test or Fisher’s exact test. A two-sided *p*-value ≤ .05 was considered significant.

Since there may be significant baseline characteristic differences between the colistin and tigecycline groups, we used propensity score matching to balance the baseline predictors for colistin or tigecycline use and mortality [23]. Propensity score matched analysis provides quasi-experimental design, which compared the outcome between two similar groups [24]. We estimated the probability of patients receiving colistin rather than tigecycline treatment with a logistic regression model, using their baseline characteristics before enrollment as variables. The fitted model was established by a stepwise variable selection procedure, with the significance levels for stay set to 0.2. Age and gender were forced to be considered in the final model. We conducted all matching on a one-to-one basis without replacement, set the caliper at 0.25, and chose the size of the caliper to be 0.0603.

For the matched cohorts, this study examined post-match balancing by using the paired *t* test or McNemar’s chi-square test [24]. We then calculated the risk difference (RD) in outcome, which included mortality, and nephrotoxicity, between the colistin group and the tigecycline group, or the average treatment effect of the treated (ATT). The data were analyzed with Stata software, version 12 (StataCorp, College Station, Texas). We used *R* 2.14.2 software (*R* Foundation for Statistical Computing, Vienna, Austria) with the Package *Matching* version 4.8-0 to conduct propensity score matching and ATT estimation.

## Results

### Patient characteristics

A total of 294 ICU-admitted patients, who were found to have MDRAB pneumonia and were treated with colistin or tigecycline, were enrolled during the study period. Among the patients, 119 received colistin and 175 received tigecycline. Table 1 presents the demographic characteristics and underlying diseases of the 294 patients with MDRAB pneumonia. There were no significant differences among the different treatment groups for age, APACHE II score, and duration of treatment. However, patients treated with tigecycline had significantly lesser underlying malignancies (1.1% vs. 28.6%, *P* < .001). The most common combination regimen used was carbapenem combination (34/294, 11.6%).

**Table 1:** **Clinical and demographic characteristics of patients with multidrug resistant *Acinetobacter baumannii *** **pneumonia treated with colistin or tigecycline**

**Characteristics**	**Total N = 294**	**Colistin N = 119**	**Tigecycline N = 175**	***p*- value**
Age	63.8 (18.5)	63.7 (19.5)	63.8 (17.9)	.95
Male	198 (67.3)	86 (72.3)	112 (64)	.16
APACHE II score	22.5 (9.1)	22.8 (9.3)	22.3 (8.9)	.68
Length of hospital stay before enrollment	32.0 (36.9)	33.2 (45.9)	31.2 (29.4)	.64
Underlying diseases				
Cardiovascular disease	47 (16.0)	17 (14.3)	30 (17.1)	.63
Diabetes mellitus	84 (28.6)	33 (27.7)	51 (29.1)	.90
Insulin use	63 (21.4)	28 (23.5)	35 (20)	.47
Chronic kidney disease	59 (20.1)	28 (23.5)	31 (17.7)	.24
Liver cirrhosis	13 (4.4)	4 (3.4)	9 (5.1)	.57
Chronic pulmonary disease	30 (10.2)	15 (12.6)	15 (8.6)	.33
Malignancy	36 (12.2)	34 (28.6)	2 (1.1)	<.001
Autoimmune	16 (5.4)	5 (4.2)	11 (6.3)	.60
Operation history	140 (47.6)	51 (42.9)	89 (50.9)	.19
Combination therapy				
Aminoglycoside	4 (1.4)	1 (0.8)	3 (1.7)	.65
Carbapenem	34 (11.6)	15 (12.6)	19 (10.9)	.65
Sulbactam	10 (3.4)	2 (1.7)	8 (4.6)	.21
Baseline creatinine (mg/dL)	1.75 (1.47)	1.66 (1.43)	1.81 (1.50)	.40
Duration of treatment	13.7 (12.5)	14.6 (13.7)	13.1 (11.6)	.31
Follow-up duration	32.4 (31.5)	33.1 (30.9)	31.9 (32.0)	.75

APACHE II, acute physiology and chronic health evaluation II.

There were no significant differences of underlying chronic kidney diseases (23.5% vs. 17.7%, *P* = .24) and baseline creatinine (1.66 vs. 1.81, *P* = .40) before treatment between the colistin and tigecycline groups. However, patients treated with colistin suffered from a significantly higher rate of nephrotoxicity (10.1% vs. 3.4%, *P* = .03). There was no significant mortality rate difference between the colistin and tigecycline groups (49.6% vs. 58.9%, *P* = .12). There was no significant mortality difference between carbapenem with the colistin combination (n = 15) and colistin without carbapenem combination (n = 104) (46.7% vs. 50.0%, *P* = .81). There was also no significant mortality difference between carbapenem with the tigecycline combination (n = 19) and tigecycline without carbapenem combination (n = 156) (42.1% vs. 60.9%, *P* = .12).

### Propensity score matching analysis

We performed a multiple variables logistic analysis to determine the significant independent variables associated with the choice of colistin rather than tigecycline (Table 2). The factors associated with using colistin were male, better baseline creatinine, insulin use, and underlying malignancy. The large number of differences among the independent variables in the colistin and tigecycline groups did not permit a definitive assessment of the effect on the outcome. Accordingly, we employed a propensity matching analysis to control for the effect of the numerous confounders.

**Table 2:** Logistic model for prediction of colistin rather than tigecycline use of the 294 enrolled patients

**Variable**	**Odds ratio**	**95% confidence interval**	***p*-value**
Age (year)	0.99	0.98 – 1.01	.69
Male	1.83	1.02 – 3.31	.04
Baseline creatinine (mg/dL)	0.76	0.57 – 1.01	.06
Chronic kidney disease × Baseline creatinine	1.42	1.09 – 1.84	.01
Insulin use	1.55	0.82 – 2.93	.18
Malignancy	40.43	9.31 – 175.52	<.001

Estimated area under the ROC curve = 0.74.

**Figure 1:**
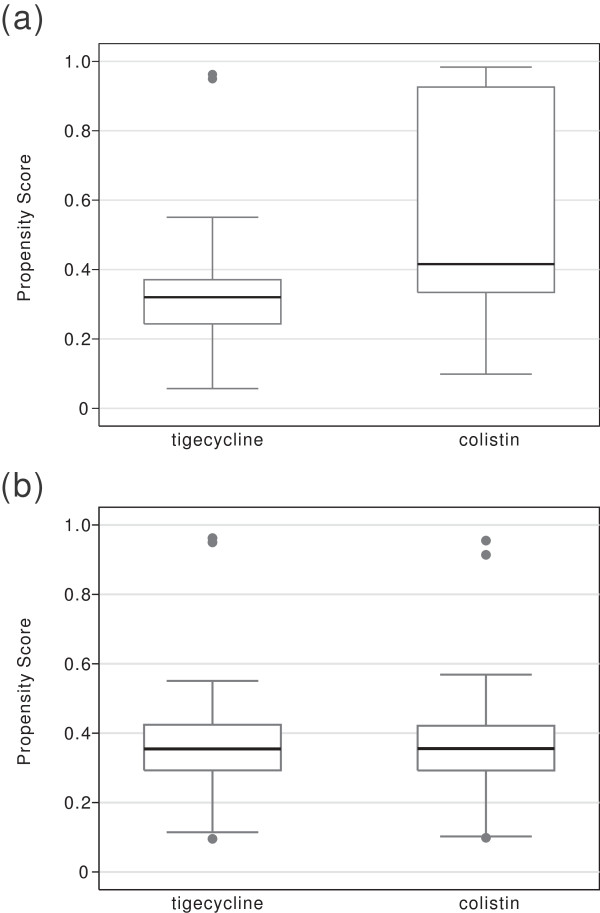
**Boxplot comparing the distribution of the *logit *** **model of the estimated propensity scores (a) before and (b) after matching patients receiving colistin with those receiving tigecycline.**

We matched 84 pairs of patients. There were no suitable tigecycline treatment controls for 35 unmatched colistin treated cases. After matching, the two groups of patients showed no significant differences of potential prognostic factors and severity of illness between them (Table 3). The propensity scores were colistin: 0.370 vs. tigecycline: 0.370, (*P* = .97); underlying malignancy (2.4% vs. 2.4%, *P* = 1.00); and insulin use (23.8% vs. 25%, *P* = .85). 163 cases had positive sputum cultures of *A. baumannii*, among them 118 sputum samples were from tracheobronchial aspirate. 36 cases had positive bronchoscopic bronchial lavage culture. The mean time (± SD) elapsed between *Acinetobacter* isolation and the beginning of antibiotics of tigecycline or colistin group is 1.9 (± 1.8) and 2 (± 1.5) days, respectively (*P* = .72). There were three (one *Chryseobacterium indologenes*, one *Escherichia coli*, and one *Enterobacter aerogenes*) polymicrobial infections in tigecycline and four (one *C. indologenes*, one *E. coli*, one *Enterobacter cloacae*, and one *Klebesiella pneumoniae*) in colistin group (*P* = .71). In patient without renal dysfunction, the mean dose of colistin used was CBA 3.0 (±0.8) mg/Kg/day. All the distributions of the estimated propensity scores were well-balanced after matching (Figure 1). We saw a significant mortality difference between the colistin and tigecycline groups (44.1% vs. 60.7%), and the tigecycline group had a significant excess mortality (RD 16.7%, 95% confidence interval (C.I.) 0.9% – 32.4%, *P* = .04). The excess mortality remained significant even after being adjusted by propensity score, age, gender, and a combination of carbapenem use (adjusted RD 16.4%, 95% C.I. 0.9% – 31.8%, *P* = .04) (Table 4). The colistin group had a higher rate of nephrotoxicity (9.5% vs. 2.4%, *P* = .05). The excess nephrotoxicity remained significant even after being adjusted by propensity score (adjusted RD 7.4%, 95% C.I. 1.2% – 13.6%, P = .02).

**Table 3:** **Clinical and demographic characteristics of the matched patients with multidrug resistant *Acinetobacter baumannii *** **pneumonia treated with colistin or tigecycline**

**Characteristics**	**Colistin N = 84**	**Tigecycline N = 84**	***p*-value**
Age	63.8 (19.8)	63.5 (17.7)	.92
Male	63 (75)	65 (77.4)	.67
APACHE II score	21.6 (9.4)	22.0 (8.8)	.99
Ventilator-associated pneumonia	58 (69.1)	60 (71.4)	.74
Early onset VAP	6 (10.3)	4 (6.7)	.38
Late onset VAP	52 (90.0)	56 (93.3)	
Length of hospital stay before enrollment	29.5 (33.7)	29.0 (30.1)	.92
Length of ICU stay before enrollment	20.2 (29.6)	18.5 (17.9)	.56
Duration of treatment	14.0 (11.2)	12.9 (9.3)	.51
Cardiovascular disease	15 (17.9)	15 (17.9)	1.00
Diabetes mellitus	24 (28.6)	27 (32.1)	.59
Insulin use	20 (23.8)	21 (25)	.85
Chronic kidney disease	22 (26.2)	22 (26.2)	1.00
Liver cirrhosis	2 (2.4)	4 (4.8)	.41
Chronic pulmonary disease	8 (9.5)	8 (9.5)	1.00
Malignancy	2 (2.4)	2 (2.4)	1.00
Autoimmune	3 (3.6)	5 (6.0)	.48
Operation history	40 (47.6)	45 (53.6)	.44
Combination therapy			
Aminoglycoside	1 (1.2)	2 (2.4)	.56
Carbapenem	9 (10.7)	9 (10.7)	1.00
Sulbactam	1 (1.2)	3 (3.6)	.32
Baseline creatine (mg/dL)	1.70 (1.40)	1.81 (1.60)	.50
Propensity score	0.370 (0.130)	0.370 (0.131)	.97

APACHE II, acute physiology and chronic health evaluation II; ICU, intensive care units; VAP, ventilator-associated pneumonia.

**Table 4:** **Multiple variable analysis of risk differences for in-hospital mortality among matched patients with *Acinetobacter baumannii *** **pneumonia**

**Variable**	**Adjusted risk differences % [95% CI]**	***p-*** **value**
Colistin vs. tigecycline	-16.4 [-0.9 to - 31.8]	.04
Propensity score	-30.3 [-85.0 to - 24.3]	.28
Age	0.3 [-0.07 to - 0.6]	.11
Male	-11.8 [-29.5 to - 5.9]	.19
Carbapenem combination	-20.8 [-45.8 to - 4.2]	.10

The MIC_50_ and MIC_90_ of colistin were 1 and 2 μg/mL, respectively. All tested isolates were susceptible to colistin. Among patients who were treated with tigecycline, only 22 have isolates available for tigecycline MIC testing. The MIC_50_ and MIC_90_ of tigecycline were 2 and 8 μg/mL, respectively. The *post-hoc* analysis analyzing the mortality showed that the excess mortality of tigecycline is significant among those with MIC >2 mg/mL (10/12 vs. 37/84, P = .01), but not for those with MIC ≦2 mg/mL (4/10 vs. 37/84, P = .81).

We rechecked the result of the propensity score matching by a different matching scenario - the overfitted model - which means all baseline factors before colistin or tigecycline use were considered in the logistic regression model without stepwise selection. The results were similar. There was excess mortality of tigecycline (RD 19.7%, 95% C.I. 2.8% – 36.6%, *P* = .02), and the colistin group had a significantly higher nephrotoxicity (RD 9.8%, 95% C.I. 1.1% – 18.6%, *P* = .03).

## Discussion

We found a significantly higher mortality rate in the tigecycline-based group and a higher rate of nephrotoxicity in the colistin-based group among the well-matched cohort. *Post-hoc* analysis showed that the mortality difference was noted in those with higher tigecycline MIC (> 2μg/mL).

There are concerns over tissue binding of colistin, poor colistin concentration in the lungs [10], and conversely tigecycline is concentrated in tissues, including lung parenchyma [25]. However, there were several meta-analysis showed excess overall mortality of tigecycline in the pooled analysis of different infection [26]– [28]. The higher mortality might be due to increased superinfections and more adverse events in the tigecycline groups [28], and an increased risk of death especially among patients with VAP [29]. The other reason for a significant treatment outcome difference may be partly due to colistin exhibiting even better *in vitro* activity to MDRAB [5]. In a previous study, Anthony et al. suggested that tigecycline MIC values in *A. baumannii* isolates may predict a clinical outcome [13]. Since our study is a retrospective design, not all isolates had *post-hoc* MIC testing. However, among the 104 preserved MDRAB isolates, the colistin susceptibility rate was 100%, whereas the tigecycline susceptibility rate was only 50.0%. Though tigecycline is concentrated in tissues, including lung parenchyma, the concentration in epithelial lining fluid is relatively low, with Cmax only 0.37 μg/mL, especially compared to high MICs of tigecycline to *A. baumannii*[25]. Though, while a high dose of tigecycline achieves a higher Cmax, and might have better clinical efficacy [30], high dose of tigecycline was not practiced at NTUH. Recent study revealed that previous colistin dosage used might be under dosing, which may possibly underestimate the effectiveness of colistin in our cohort [31].

Combination therapy for MDRAB infection is controversial. The combination of tigecycline with carbapenem showed a comparable outcome than tigecycline monotherapy [12], while the combination of colistin with carbapenem showed a less favorable outcome than colistin monotherapy [32]. According to Taiwanese regulations (Regulations for National Health Insurance Reimbursement), only few cases in out cohort had received tigecycline or colistin combination therapy. Although our study did not support the effectiveness of combination therapy for MDRAB pneumonia, further large sample sized prospective studies are warrant to illustrate the issue.

Colistin presented nephrotoxicity in around 6% of treated patients [8]. However, in the meta-analysis of the two arms study, Florescu et al. reported no significant higher rates of nephrotoxicity in the colistin group than that in the comparator group (Odds ratio 1.14, 95% C.I. 0.59 – 2.20, *P* = .69). The difference may be due to the fact that the baseline characteristics of the colistin and comparator groups might be different especially in the observational studies, but they were not adjusted in the meta-analysis. In our study we showed borderline significant excess nephrotoxicity of 7.1% of the colistin group compared to tigecycline group (*P* = .05) for the baseline characteristic comparable cohorts. Thus, for patients with predisposing factors such as nephrotoxic drugs, chronic kidney diseases, and hypovolemia, we should carefully monitor the renal function during colistin use [11].

This study has several limitations. First, the majority of the patients were diagnosed using qualitative sputum culture, which might result in over-diagnosis of pneumonia. Hence, the difference of the effectiveness between tigecycline and colistin might be underestimated. Second, there are difficulties inherent in the design of any retrospective matched cohort study. We may have missed some unobserved confounders that were unbalanced, even though the fitness of our prediction models appear to be good (area under ROC: 0.74), and the result is robust between different matching scenarios. In order to avoid any possible misclassification bias in the retrospective design, a hard end point - mortality - is used rather than clinical improvement. Third, since there were no MIC interpretation criteria of tigecycline against *A. baumannii*[2], our microbiology lab had no routine antimicrobial susceptibility test of tigecycline. Thus not all isolates had tigecycline susceptibility tested. Lastly, in our lab, the MIC testing of colistin and tigecycline were uniformly performed using agar dilution. The result of broth microdilution might be affected by whether fresh medium was used or Oxyrase was added, but agar dilution might not [33,34]. However, limited study showed that the MIC obtained by agar dilution is lower than that obtained by broth dilution techniques [33].

## Conclusions

In patients with possible nephrotoxic factors, their renal function during colistin use should be carefully monitored. Our data disfavors the use of tigecycline-based treatment in treating MDRAB pneumonia when tigecycline and colistin susceptibilities are unknown, since choosing tigecycline-based treatment might result in higher mortality. The excess mortality of tigecycline-based group may be related to higher MIC of tigecycline (> 2 μg/mL). There is an urgent need to establish the interpretation criteria of tigecycline for MDRAB infection. The effectiveness of tigecycline use in those with low tigecycline MIC in the era of emerging MDRAB warrants further study.

## Abbreviations

APACHE: Acute Physiology and Chronic Health Evaluation; ATT: The average treatment effect of the treated; CBA: Colistin base activity; C.I.: Confidence interval; CLCr: Calculated creatinine clearance; CLSI: Clinical and Laboratory Standards Institute; EUCAST: European Committee on Antimicrobial Susceptibility Testing; ICU: Intensive care units; MDRAB: Multi-drug resistant *Acinetobacter baumannii*; MIC: Minimum inhibitory concentration; NTUH: National Taiwan University Hospital; RD: Risk difference; SD: Standard deviation.

## Competing interests

The authors declare that they have no competing interests.

## Authors’ contributions

YCC, and CYC reviewed the medical records and drafted the manuscript; YCC, HYS, and WJT analyzed and interpreted the data. WHS designed and oversaw the study, analyzed and interpreted the data, and revised the manuscript. YCC and SCC revised the manuscript. All authors have read and approved the manuscript for publication.

## Pre-publication history

The pre-publication history for this paper can be accessed here:

http://www.biomedcentral.com/1471-2334/14/102/prepub
